# Exploring Response Patterns to Motivational Messages Supporting Physical Activity: Latent Class Analysis

**DOI:** 10.2196/91917

**Published:** 2026-08-19

**Authors:** Chihiro Moriishi, Takeyuki Oba, Keisuke Takano, Kentaro Katahira, Kenta Kimura

**Affiliations:** 1 Human Informatics and Interaction Research Institute National Institute of Advanced Industrial Science and Technology Tsukuba, Ibaraki Japan; 2 Faculty of Letters Hosei University Chiyoda-ku, Tokyo Japan

**Keywords:** behavior change technique, clustering algorithm, digital health, physical activity, text message

## Abstract

**Background:**

In mobile health care, text messages play an important role in improving physical activity. Recent studies have developed message banks based on theories, including the behavior change technique (BCT) taxonomy. However, little evidence is available for individual differences (ie, who responds to which BCTs presented in messages), which is crucial for optimizing message delivery.

**Objective:**

We investigated how individuals perceive messages supporting physical activity and what clusters of individuals are identified by their responses.

**Methods:**

Japanese-speaking adults (n=2859; mean age 54.5, SD 17.5 years; 1486 women) were presented with messages conveying different BCTs and rated how motivational each message was. The motivation ratings were subjected to latent class analysis to identify clusters of individuals per motivation rating.

**Results:**

A total of 7 clusters were identified. Two clusters gave consistently high ratings across BCT types; one showed a general receptivity to all messages, while the other showed a clearer preference for information-based BCTs. Two clusters showed moderate ratings, both preferring information about consequences but differing in their additional preferences for goal setting vs rewards. Two clusters gave overall low ratings and typically included less active individuals in the preaction stages, both showing a preference for information-based BCTs. The remaining cluster showed the greatest variability in BCT preferences, with the strongest preference for salience of consequences.

**Conclusions:**

These results highlight individual differences in perceived motivations across BCTs, informing what BCTs should be prioritized in delivery. The practical implications for message tailoring are also discussed.

## Introduction

### Background

The rapid growth of mobile technology enables remote, interactive, and timely access to relevant health information, expanding the applicability of health behavior change interventions to promote physical activity (PA) [1]. A popular intervention modality is the use of SMS text messages to deliver educational materials, health care tips, individual goals and achievements, and other motivational information. Text message interventions (TMIs) are considered a promising form of mobile health (mHealth) intervention for PA [2] because smartphone ownership is very high and the implementation cost is relatively low (eg, an estimated average cost per message is US $0.10) [3]. Furthermore, messages allow for quick and easy access to necessary information and require little or no effort for information search, which helps message recipients maintain their interest in and commitment to health behavior goals [4]. Another important advantage is that messages can be tailored to recipients’ demographic characteristics and psychosocial attributes, or even personalized for individual recipients (eg, to include their names in message headers) to enhance message acceptability and the efficacy of an intervention [5]. TMIs can also be scaled up as ecological momentary or just-in-time adaptive interventions [6] if messages are designed to target a particular moment, such as when a recipient is most likely to accept and respond to messages.

A literature review [7] identified 6 TMI trials targeting PA, of which 3 reported statistically significant effects of TMIs. A meta-analysis [8] synthesized the results of 10 trials, most of which assessed self-reported PA as an outcome, and estimated an effect size of Cohen *d*=0.50. Another meta-analysis [2] of 13 trials targeting objectively measured PA concluded that TMIs lead to significantly greater postintervention steps (*d*=0.38), whereas the effect on moderate to vigorous PA was smaller and marginal (*d*=0.31). Notably, both meta-analyses documented moderate to high heterogeneity in efficacy [2,8], which can be at least partly attributed to the variability in TMI delivery and implementation. Therefore, exploring how TMIs can be optimized is important, particularly to clarify to whom and what messages should be delivered.

Researchers have developed message banks to address this challenge. Several different messages are registered in a bank, from which practitioners and app-implemented algorithms can select the appropriate ones to deliver to their client users. Text messages take various forms in terms of content and tone, depending on the purpose of the TMI (eg, providing education, enhancing motivation, delivering feedback related to goals, and sending reminders for participants’ PA plans) [2]. The development of messages is also based on various sources, such as feedback from experts and patients [9], clinical guidelines [10], and reviews of previous studies [11].

Messages are typically tailored to a target population based on their demographic and psychosocial characteristics. Pathak et al. [12] developed messages for low-income minority individuals with comorbid diabetes and depression by lowering the reading level (below the sixth-grade level) and ensuring cultural and linguistic appropriateness. Feedback from message recipients is also an important source for tailoring messages, which has been suggested to improve the ease of understanding and acceptability of the delivered messages (eg, for patients with chronic obstructive pulmonary disease [9] and coronary heart disease [13]). Moreover, message content and tone can vary across individuals at different stages of behavior change, particularly when integrated into a treatment or intervention. Prior studies have used tailoring focused on recipients’ gender and PA levels [14], lifestyle habits among adults with type 2 diabetes (eg, individuals’ dietary behavior, PA, and medication) [15], and goal progress among older adults at risk of cardiovascular disease [11]. However, researchers have noted the lack of theoretical underpinnings for several message banks and argued that the design and validation processes are not always transparent [16]. These circumstances critically limit the opportunities for replicating and reproducing TMIs and prevent fair evaluations of message quality [17,18]. Furthermore, having a link to theories appears to directly affect the efficacy of digital interventions. For example, 1 meta-analysis showed larger effects of theory-based interventions than those not based on a specific theory ([19]; however, see also [5,20]). An exceptional study is MacPherson et al [18], who developed messages to deliver in a diabetes prevention program using the behavior change wheel (BCW) [21]. The BCW is an integrative framework used to guide the development of behavior change interventions [22] and can be a theoretical basis for researchers and practitioners to select the intervention approaches or behavior change techniques (BCTs) that are most suitable for addressing individuals’ specific barriers and facilitators [18]. BCTs have been structured and numbered in the form of a taxonomy [21] to which 93 different types of BCTs (classified into 16 categories) are registered, such as goal setting—behavior (1.1) and problem-solving/coping planning (1.2). In their message-development process, MacPherson et al [23] identified 43 BCTs relevant for PA (and dietary) behavior change adherence, encompassing barriers and facilitators to engage in target behaviors. For the identified BCTs, messages were developed and refined based on feedback from program coaches and participants. Therefore, each message is tagged with a specific BCT-taxonomy label while maintaining clarity and acceptability for recipients [18]. Example messages include “Think about what exercise goal you want to make today, if any,” tagged with the BCT labels of goal setting—behavior (1.1) and outcome (1.3), and “Sometimes we need the support of others to help us stick to our goals. Think about who in your life can help you stick to your exercise plan,” labeled as social support—emotional (3.3) and unspecified (3.1). The message bank comprises BCTs that are most frequently implemented in PA intervention, including goal setting, social support, self-monitoring of behavior, and instruction on how to perform the behavior, which have been commonly identified in systematic reviews and meta-analyses on mHealth interventions for various populations (eg, adults, children, and patients with a specific disease [24-31]).

However, as MacPherson et al [18] acknowledged, which specific messages (or BCTs offered in messages) are more effective than others remains unclear. As noted above, although the BCW serves as a foundation for interventions using BCTs, it does not clearly specify which BCTs work best, for whom, in which contexts, and delivered by what means [32]. Although common BCTs, such as goal setting and self-monitoring of behavior, are frequently used in interventions, they are not necessarily perceived as helpful or motivational by all recipients. Thus, an important evidence gap is that individual differences in terms of who finds which messages helpful remain understudied. These issues are of practical importance when tailoring message delivery because knowing which messages should be delivered to whom is an important step toward achieving precision health [33]. To address these individual differences, the Behavior Change Resource Model (BCRM) may be a useful framework [34]. BCRM assumes that BCTs work by acting on individuals’ resources and proposes a behavior change framework based on these resources. It distinguishes three types of resources: facilitating (providing external resources), boosting (building and strengthening internal reflective resources), and nudging (activating affective resources). These 3 resources partly correspond to the capability, opportunity, motivation-behavior (COM-B) model, which is central to the BCW [35], but the 2 frameworks have different roles. COM-B classifies the conditions necessary for behavior. In contrast, BCRM explains how resources are generated and activated in the brain’s motivational and reward systems and describes how BCTs act on these resources, based on neurobiological mechanisms. In this way, because BCRM classifies BCTs according to the resources they target, differences in BCT preferences across clusters may reflect differences in the resources each cluster needs or prioritizes. Michaelsen and Esch [34] also suggest identifying the resources needed to move to the next stage of behavior change and selecting BCTs accordingly. For example, individuals in the early stages of behavior change may lack motivation and may prefer BCTs categorized as nudging, which do not require deliberate effort. In contrast, individuals who are already performing the behavior may prefer BCTs categorized as boosting, which involve intentional effort.

### Objectives

This study investigated how individuals perceive BCT-tagged messages for improving PA. We focused on individual differences in participants’ responses to different BCTs recommended in the messages. Specifically, we assumed that different individuals would find different BCTs to be motivational. Therefore, we used a clustering approach to identify groups of people who showed similar responses to presented messages. Through this analysis, we aimed to determine how many response patterns (ie, clusters) should be assumed in a sample and which messages can be delivered together to a particular group of recipients. Moreover, we explored how frequently implemented BCTs [24-31] would manifest in these clusters. We expected that while most clusters would perceive goal setting and other common BCTs as motivational, some clusters might not prefer them. The analysis was conducted using cross-sectional survey data, in which participants rated how motivational each BCT message was in enhancing PA. Messages were adapted from the bank of MacPherson et al [18], to which we added (1) messages localized for the target population of this study (Japanese-speaking adults) to indicate the exact information source such as a health guideline of the country, and (2) messages framed with gain and loss focuses ([36]: *gain framing*, focusing on benefits of a behavioral alternative, and *loss framing*, emphasizing the associated costs of a behavior) [36-38]. Although framing is not a dimension well integrated into the BCW or BCT taxonomy, it has been applied to behavior change based on prospect theory, which suggests that individuals respond differently depending on whether information is presented from a cost (loss framing) or benefit (gain framing) perspective [36]. However, in the context of PA, it remains unclear which type of framing is more effective [39]. Therefore, examining which individuals are more likely to prefer each type of framing, in combination with BCT preferences, may help inform more precise message delivery design. We exploratorily examined how it appeared across clusters of message response types (eg, a cluster might find loss framing and goal setting more motivational than other message types).

## Methods

### Participants

Participants (N=2862) were recruited from a database of a survey firm, in which >1.3 million individuals (all residents in Japan) had been registered as potential participants. This study was part of longitudinal surveys started in 2022, and the sampling for this study was conducted in November 2024. The overarching project had multiple purposes, for example, to investigate motivational and environmental factors that influence PA [40] and the prevalence of mHealth tool use among Japanese-speaking adults [41,42]. For this study, invitations were sent to the participants who completed the initial, main survey of the overarching project (n=20,611 [43]). Quota sampling was used to ensure that the data covered each age group and gender. The sample size was determined arbitrarily—we aimed to sample around 100 participants for each age group of 5-years interval for each gender. No missing values were recorded as the survey platform required participants to respond to each item to complete the survey. Participants were allowed to stop responding in the middle of the survey, but in that case, no record was created in the database. Data from 3 participants were deemed unreliable and excluded from analysis (2 reported a height of <100 cm and 1 reported a weight of <10 kg). The final data included 2859 participants. We adhered to the STROBE (Strengthening the Reporting of Observational Studies in Epidemiology) statement [44] in conducting and reporting this study.

### Ethical Considerations

The study protocol was approved by the Ethics Committee of the National Institute of Advanced Industrial Science and Technology (approval ID: 2022-1279). Participants provided informed consent prior to responding to the survey. Participants were compensated with a voucher for online shopping after the completion of the survey. All data were collected via an online survey company and provided to the research team in anonymized form, such that individual participants could not be identified. The dataset was stored on a password-protected computer accessible only to the research team.

### Measures

#### Message and Motivation Rating

Each participant rated 83 messages, 72 of which were adapted and translated from the bank of MacPherson et al [18], which was originally developed for a type 2 diabetes prevention program conducted in Canada. Messages that included program-specific references or mentions of specific service names were excluded (eg, “Remember the exercise you did at the YMCA with your coach? Try to reach those same target heart rate zones on your own!”). After translation, the content validity of the messages was confirmed by the second and third authors. For the remaining messages, 3 were gain-framed, 3 were loss-framed, and 5 were localized with explicit mentions of the health guidelines and authorities in Japan (as credible sources; for the specific messages, see Table S1 in Multimedia Appendix 1). Messages were contextualized in terms of digital health, with the instructions indicating that messages were intended to be delivered via AI or a chatbot implemented through a smartphone app supporting PA and exercise. The instructions are as follows:

Below are messages designed to support and motivate exercise and PA. These messages are intended for a broad audience, including those who do not have exercise habits yet and those who already maintain an exercise routine. Please read these messages and rate how motivated you would feel if you received them. The intended context is that individuals seek to change their lifestyle habits to maintain or improve their health, and they received these messages in their daily lives. Please assume that the sender of the messages is a chatbot or AI implemented in an exercise or training app.

Each message was rated on a 5-point scale (1=not at all; 5=very much). The number of BCT labels that appeared in the message bank was not balanced (Table 1). The most frequent label was information about health consequences (5.1), tagged for 14 messages, followed by credible source (9.1), for 11 messages.

**Table 1:** Number of messages per behavior change technique (BCT) and framing label and mean motivation ratings (n=2859)^a^.

BCT/framing		Messages, n (%)	Ratings, mean (SD)
Goals and planning (1)			
	Goal setting—behavior (1.1)	1 (0.7)	2.41 (1.06)
	Problem solving (1.2)	3 (2.2)	2.29 (1.05)
	Goal setting—outcome (1.3)	3 (2.2)	2.41 (1.11)
	Action planning (1.4)	7 (5.1)	2.32 (1.07)
	Review outcome goals (1.7)	2 (1.5)	2.39 (1.05)
Feedback and monitoring (2)			
	Self-monitoring of behavior (2.3)	4 (2.9)	2.25 (1.06)
Social support (3)			
	Social support—unspecified (3.1)	5 (3.6)	2.32 (1.09)
	Social support—practical (3.2)	1 (0.7)	2.40 (1.14)
Shaping knowledge (4)			
	Instruction on how to perform the behavior (4.1)	4 (2.9)	2.40 (1.07)
	Behavioral experiments (4.4)	1 (0.7)	2.25 (1.03)
Natural consequences (5)			
	Information about health consequences (5.1)	14 (10.2)	2.42 (1.08)
	Salience of consequences (5.2)	1 (0.7)	2.59 (1.06)
	Information about social and environmental consequences (5.3)	1 (0.7)	2.64 (1.12)
	Monitoring of emotional consequences (5.4)	1 (0.7)	2.29 (1.13)
	Information about emotional consequences (5.6)	2 (1.5)	2.48 (1.11)
Comparison of behavior (6)			
	Social comparison (6.2)	4 (2.9)	2.38 (1.07)
Associations (7)			
	Prompts/cues (7.1)	3 (2.2)	2.24 (1.08)
Repetition and substitution (8)			
	Behavioral practice/rehearsal (8.1)	1 (0.7)	2.32 (1.06)
	Behavioral substitution (8.2)	4 (2.9)	2.39 (1.05)
	Habit formation (8.3)	1 (0.7)	2.32 (1.06)
	Habit reversal (8.4)	3 (2.2)	2.40 (1.06)
	Graded tasks (8.7)	2 (1.5)	2.55 (1.04)
Comparison of outcomes (9)			
	Credible source (9.1)	11 (8)	2.35 (1.07)
	Pros and cons (9.2)	1 (0.7)	2.40 (1.04)
Reward and threat (10)			
	Nonspecific reward (10.3)	7 (5.1)	2.45 (1.07)
	Nonspecific incentive (10.6)	1 (0.7)	2.41 (1.07)
	Self-incentive (10.7)	2 (1.5)	2.41 (1.08)
	Self-reward (10.9)	7 (5.1)	2.45 (1.07)
Regulation (11)			
	Reduce negative emotions (11.2)	2 (1.5)	2.36 (1.09)
	Conserving mental resources (11.3)	2 (1.5)	2.36 (1.09)
Antecedents (12)			
	Restructuring the physical environment (12.1)	2 (1.5)	2.35 (1.05)
	Restructuring the social environment (12.2)	1 (0.7)	2.35 (1.06)
	Distraction (12.4)	3 (2.2)	2.37 (1.09)
Identity (13)			
	Identification of self as role model (13.1)	3 (2.2)	2.33 (1.11)
	Framing/reframing (13.2)	3 (2.2)	2.38 (1.05)
Scheduled consequences (14)			
	Reward approximation (14.4)	5 (3.6)	2.46 (1.08)
	Reward completion (14.5)	1 (0.7)	2.41 (1.05)
Self-belief (15)			
	Verbal persuasion about capability (15.1)	4 (2.9)	2.43 (1.09)
	Focus on past success (15.3)	4 (2.9)	2.44 (1.10)
	Self-talk (15.4)	3 (2.2)	2.31 (1.06)
Covert learning (16)			
	Imaginary reward (16.2)	1 (0.7)	2.36 (1.10)
Gain framing		3 (2.2)	2.40 (1.07)
Loss framing		3 (2.2)	2.35 (1.06)

^a^Some messages had multiple BCT labels.

#### PA Measure

Subjective PA was assessed using the International Physical Activity Questionnaire-Short Form (IPAQ-SF) [45,46]. The IPAQ-SF asks respondents to report the number of days and duration spent on each of the following three PA dimensions over an average week: (1) walking, (2) moderate-intensity activity, and (3) vigorous-intensity activity. Weekly minutes of PA were calculated and transformed into metabolic equivalents (METs; METs-hour/week) to represent total PA levels. The IPAQ-SF originally included items on sedentary time, which were not used in this study.

#### Stage-of-Change for PA

Stages of change (SoC) for PA were assessed using the SoC questionnaire [47-50]. Each participant indicated the most applicable statement from the following five statements: “I currently do not exercise and do not intend to start exercising in the future” (Precontemplation); “I currently do not exercise but I am thinking about starting to exercise in the next six months” (Contemplation); “I currently exercise some, but not regularly” (Preparation); “I currently exercise regularly, but have only begun doing so within the last six months” (action); and “I currently exercise regularly and have done so for longer than six months” (Maintenance). The instructions explicitly defined regular exercise as exercising twice or more per week for 20 minutes or longer on each occasion.

### Statistical Analysis

Latent class analysis (LCA) was used to identify groups of individuals (ie, clusters) who gave similar ratings to the presented messages [51]. LCA is a type of finite mixture modeling that represents clusters in the form of categorical latent variables estimated from categorical observed variables. We performed LCA on dichotomized motivation ratings (with values coded based on whether they were above or below the overall mean) using maximum likelihood estimation. LCA computes the probabilities for each observation to belong to each cluster; therefore, we attributed each participant to the cluster to which they were most likely to belong [52]. To determine the optimal number of clusters, models were estimated with 2-14 clusters, and model fit was evaluated using the Bayesian Information Criterion (BIC). The characteristics of each identified cluster were inspected based on the (1) mean motivation ratings for each message in each cluster, (2) mean motivation ratings per BCT label in each cluster, and (3) standardized mean ratings per BCT label within each cluster. Within-cluster standardization helped interpret which BCTs each cluster perceived as more (or less) motivational.

## Results

### Demographic Characteristics and Descriptive Statistics

Table 2 summarizes the participants’ demographic characteristics. More than half of the participants were married and had at least 1 child. Most had graduated from high school, and household income was distributed around JPY 2-6 million (US $12,600-US $37,900), which is comparable to the population distribution in Japan (average JPY 5.45 million [US $34,400]). On average, participants were more active than the recommended level (23 METs-hours/week) by the authorities, and approximately one-third reported being in the maintenance stage. Table 1 presents the mean motivation ratings for each BCT label. Information about social and environmental consequences (5.3) was rated as the most motivational, followed by salience of consequences (5.2). The least motivational messages were prompts/cues (7.1), self-monitoring of behavior (2.3), and behavioral experiments (4.4).

**Table 2:** Demographic characteristics and descriptive statistics (n=2859).

Variable			Value
Age (years), mean (SD)			54.49 (17.52)
Women, n (%)			1486 (51.98)
BMI, mean (SD)			21.77 (3.7)
Married, n (%)			1723 (60.27)
One or more children, n (%)			1636 (57.22)
Job, n (%)			1685 (58.94)
Education level, n (%)			
	Middle/high school	926 (32.39)	
	Two-year college or vocational school	627 (21.93)	
	University or above	1292 (45.19)	
	Other	14 (0.49)	
Household income (JPY; JPY 1=US $0.0063), n (%)			
	<2 million	269 (9.41)	
	2-4 million	659 (23.05)	
	4-6 million	537 (18.78)	
	6-8 million	363 (12.7)	
	8-10 million	211 (7.38)	
	10 million or above	252 (8.81)	
	No response or unknown	568 (19.87)	
PA^a^ (METs^b^-hour/week), mean (SD)			35.84 (56.21)
Stage of change, n (%)			
	Precontemplation	767 (26.83)	
	Contemplation	498 (17.42)	
	Preparation	519 (18.15)	
	Action	95 (3.32)	
	Maintenance	980 (34.28)	

^a^PA: physical activity.

^b^MET: metabolic equivalent.

### Latent Class Analysis

**Figure 1:**
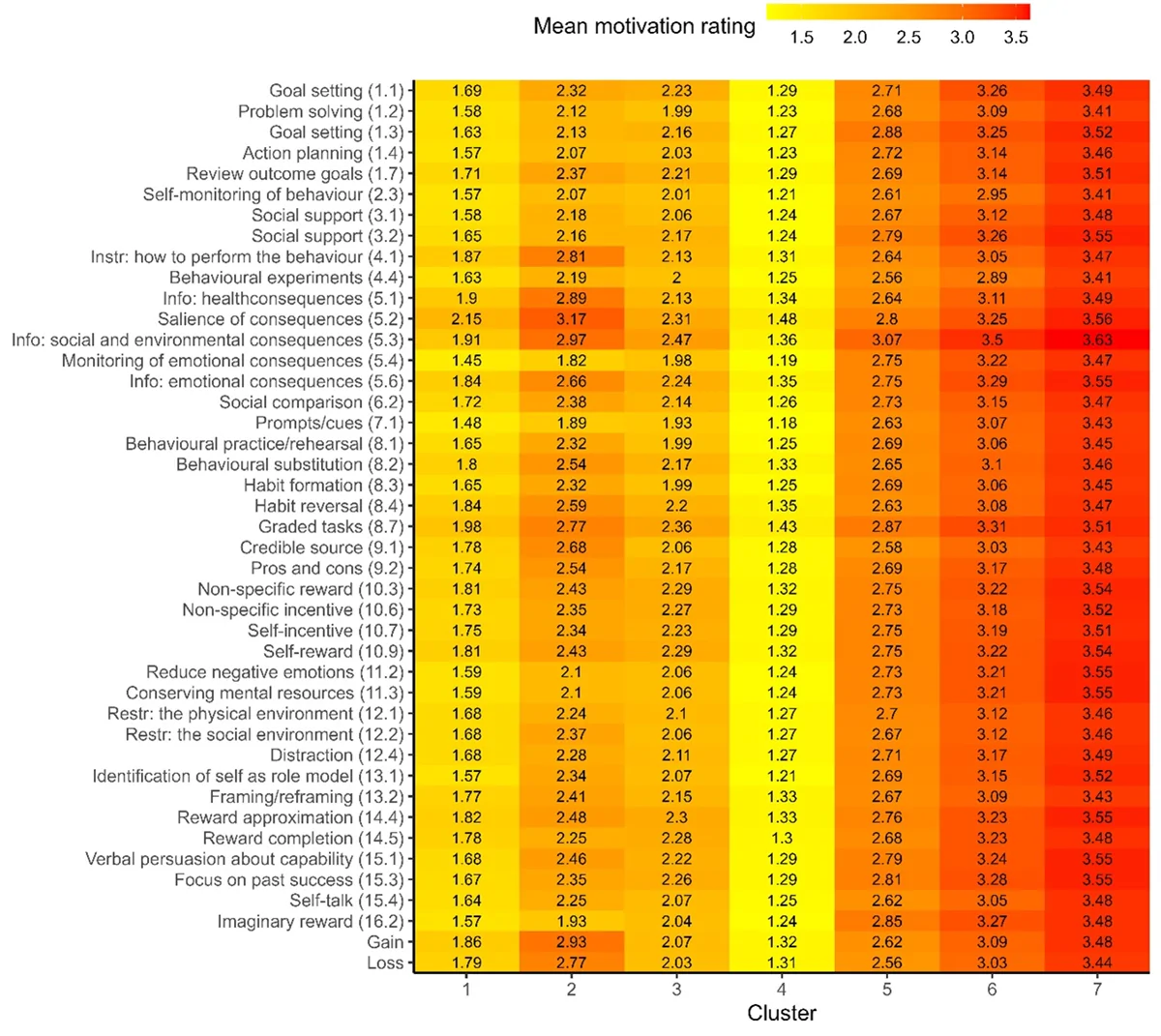
Mean motivation ratings per behavior change technique and cluster.

The LCA identified 7 clusters as the best solution that achieved the minimum BIC (5 clusters=198,459, 6 clusters=197,399, 7 clusters=197,350, 8 clusters=197,413, and higher for the larger number of clusters). The entropy for the 7-cluster solution was 0.943, indicating that the classification accuracy was sufficient. Among the identified 7 clusters, cluster 7 had the highest motivation ratings for each message, whereas cluster 4 had the lowest ratings (Figure 1). The other clusters fell between clusters 7 and 4, although cluster 2 showed the largest variance across messages, suggesting that it had the most selective preference for different messages.

### Cluster Characteristics

**Figure 2:**
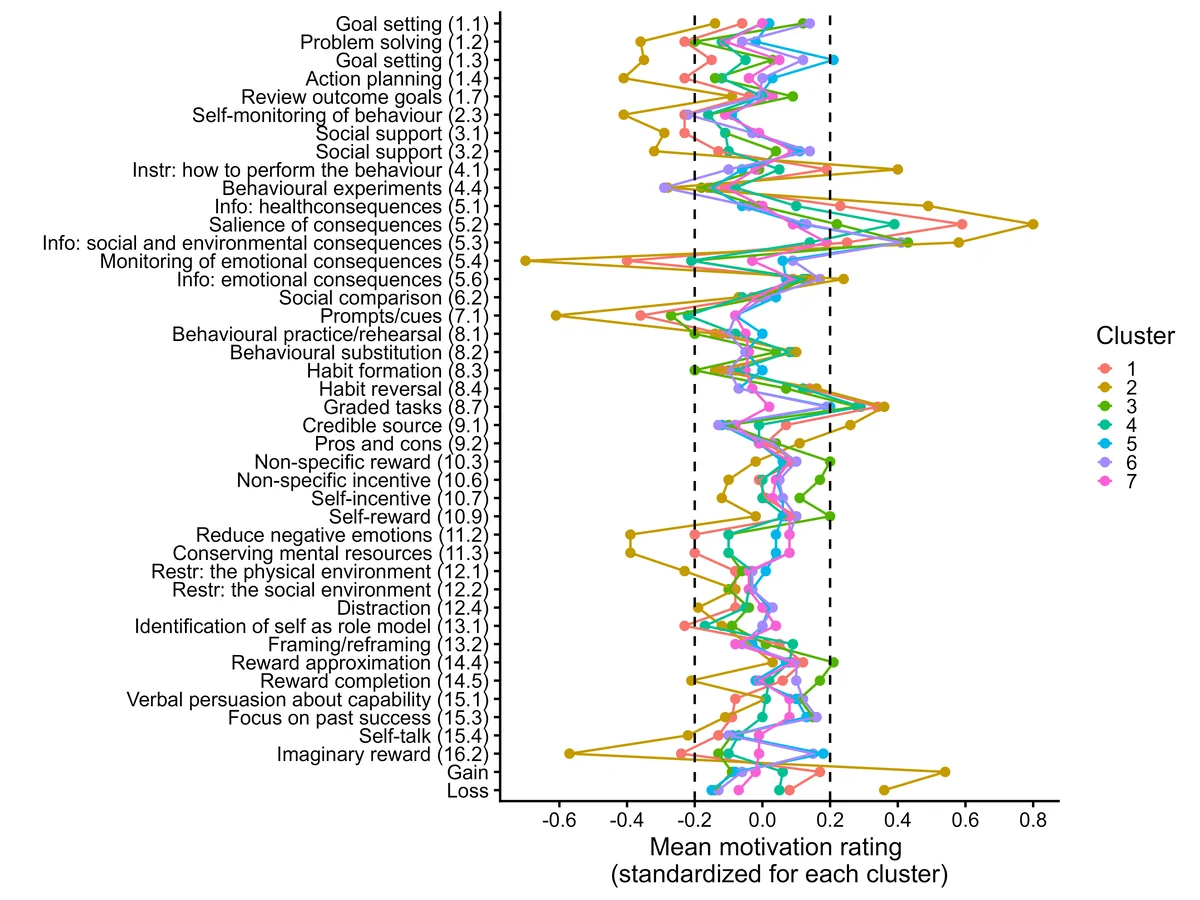
Mean motivation ratings standardized within each cluster.

We calculated the mean motivation ratings per BCT (Figure 1) and standardized them within each cluster to visualize the rating patterns across clusters (Figure 2). Statistical tests did not help interpret between- and within-cluster differences because of the enormous number of possible paired comparisons between BCTs and clusters. Therefore, we focused on (1) the BCTs with the highest and lowest standardized mean motivation ratings within each cluster, (2) the BCTs whose within-cluster standardized mean exceeded the reference threshold in that cluster but not in any other cluster. A within-cluster standardized mean of 0.20 was used as a reference threshold to identify BCTs with meaningful differences in ratings, corresponding to a small effect size based on Cohen’s (1988) benchmarks (small: *d*=0.20; medium: *d*=0.50; large: *d*=0.80) [53].

Cluster 7 gave the highest ratings to any BCTs, with almost no preference for a particular BCT. No BCTs had within-cluster standardized mean values greater than 0.20 or less than –0.20. Cluster 6 also showed high ratings for most BCTs, with most mean motivation ratings above 3.0, but exhibited a clearer preference pattern than cluster 7. Specifically, it showed the highest ratings for information about social and environmental consequences (5.3: within-cluster standardized mean 0.41) and the lowest for behavioral experiments (4.4: within-cluster standardized mean –0.29). Clusters 5 and 3 showed overall moderate ratings for BCTs, with most mean motivation ratings between 2.0 and 3.0. Cluster 5 showed the highest rating for information about social and environmental consequences (5.3: within-cluster standardized mean 0.41), and no BCTs were rated low enough to exceed the reference threshold (no BCTs with a within-cluster standardized mean below –0.20). Goal setting (1.3: within-cluster standardized mean 0.21) exceeded the reference threshold only in this cluster. Similarly, cluster 3 showed the highest ratings for information about social and environmental consequences (5.3: within-cluster standardized mean 0.43) and the lowest for prompts/cues (7.1: within-cluster standardized mean –0.27). Nonspecific rewards (10.3: within-cluster standardized mean 0.20), self-reward (10.9: within-cluster standardized mean 0.20), and reward approximation (14.4: within-cluster standardized mean 0.21) exceeded the reference threshold only in this cluster. Clusters 4 and 1 showed overall low ratings for BCTs, with most mean motivation ratings between 1.0 and 2.0. Cluster 4 showed the highest ratings for salience of consequences (5.2: within-cluster standardized mean 0.39) and the lowest for prompts/cues (7.1: within-cluster standardized mean –0.22). Cluster 1 showed the highest ratings for salience of consequences (5.2: within-cluster standardized mean 0.59) and the lowest for monitoring of emotional consequences (5.4: within-cluster standardized mean –0.40). Identification of self as role model (13.1: within-cluster standardized mean –0.23) exceeded the negative reference threshold only in this cluster. Finally, cluster 2 showed the strongest differentiation in preferences for BCTs. It showed the highest ratings for salience of consequences (5.2: within-cluster standardized mean 0.80) and the lowest for monitoring of emotional consequences (5.4: within-cluster standardized mean –0.70). BCT groups related to goals and planning, feedback and monitoring, and social support did not exceed the positive reference threshold in this cluster, whereas instruction on how to perform the behavior (4.1: within-cluster standardized mean 0.40) exceeded it. High ratings for both gain- and loss-framed messages were also observed (gain: within-cluster standardized mean 0.54; loss: within-cluster standardized mean 0.36).

Among the BCTs commonly used in interventions (ie, goal setting, social support, self-monitoring of behavior, and instruction on how to perform the behavior), goal setting (1.3) exceeded the positive reference threshold only in cluster 5 (within-cluster standardized mean 0.21), whereas it exceeded the negative reference threshold only in cluster 2 (within-cluster standardized mean –0.35). Instruction on how to perform the behavior (4.1) exceeded the positive reference threshold only in cluster 2 (within-cluster standardized mean 0.40). Self-monitoring of behavior (2.3) exceeded the negative reference threshold in clusters 1, 2, and 6, as did social support (3.1) in clusters 1 and 2, and social support (3.2) in cluster 2.

Table 3 presents the demographic characteristics, self-reported PA levels, and SoC for each cluster. Clusters 7 and 6 were the most male-dominant (191/364, 52% and 288/514, 56%), whereas cluster 2 was the oldest (mean age 63.8 years) among the 7 clusters. BMI showed no significant between-cluster differences. Clusters differed significantly in PA level (*F*_6, 2852_=11.39; *P*<.001); however, the omnibus effect size was small (ηp^2^=.02), indicating that the practical magnitude of between-cluster differences in PA was limited despite statistical significance arising from the large sample. Pairwise comparisons were therefore interpreted with this small overall effect in mind, using the same benchmarks as reference points for effect size interpretation (small: *d*=0.20; medium: *d*=0.50; large: *d*=0.80). Descriptively, cluster 7 showed the highest PA level, followed by cluster 2; this difference was negligible in magnitude (*d*=0.152). Cluster 6 and cluster 7 differed by a similarly small margin (*d*=0.200), while cluster 6 and cluster 2 showed almost no difference (*d*=0.048). Cluster 4 showed the lowest PA level, and the remaining clusters (1, 3, and 5) fell in between, with negligible differences among them (ds<0.05). Approximately half (43%-53%) of clusters 7, 6, and 2 were in the maintenance stage. The least active cluster was cluster 4, in which >80% of the individuals were in the preaction (precontemplation-preparation) stages. In other clusters (1, 3, and 5), the proportions were similar across stages, except for the action stage. Although pairwise comparisons did not detect any significant differences in PA levels between adjacent clusters (eg, cluster 7 vs 2, see Tables S2 and S3 in Multimedia Appendix 1), the high-rating clusters (clusters 7, 6, 5, and 2) had significantly higher PA levels than the low-rating clusters (clusters 1, 3, and 4); t_2857_=6.062; *P*<.001; *d*=0.227). Similarly, the proportion of individuals in the maintenance stage was higher in high-rating clusters than in low-rating ones (odds ratio 2.089, 95% CI 1.78-2.45; *P*<.001).

**Table 3:** Demographic characteristics of the latent class analysis–identified clusters.

Variable		Cluster 1 (n=431)	Cluster 2 (n=142)	Cluster 3 (n=338)	Cluster 4 (n=547)	Cluster 5 (n=523)	Cluster 6 (n=514)	Cluster 7 (n=364)	Cluster difference							
									Test statistics		Effect size (ηp^2^)		*P* value			
Age (years), mean (SD)		59.25 (15.41)	63.75 (13.18)	56.69 (17.24)	53.63 (15.95)	49.86 (17.9)	50.44 (19.15)	56.84 (17.42)	26.08 (6, 2852)^a^		0.05		<.001			
Women, n (%)		270 (62.65)	79 (55.63)	189 (55.92)	290 (53.02)	259 (49.52)	226 (43.97)	173 (47.53)	40.11 (6)^b^		N/A^c^		<.001			
BMI (kg/m^2^), mean (SD)		21.56 (4.00)	21.72 (3.40)	21.96 (3.84)	21.95 (4.05)	21.74 (3.38)	21.63 (3.40)	21.85 (3.56)	0.74 (6, 2852)^a^		0.00		.62			
PA^d^ (METs^e^-hours/week), mean (SD)		32.22 (48.13)	43.48 (58.13)	34.44 (59.94)	23.06 (53.21)	34.94 (60.30)	40.81 (48.00)	51.93 (64.46)	11.39 (6, 2852)^a^		0.02		<.001			
Stage of change, n (%)										380.25 (24)^b^		N/A		<.001		
	Precontemplation	130 (30.16)	17 (11.97)	79 (23.37)	295 (53.93)	123 (23.52)	76 (14.79)	47 (12.91)								
	Contemplation	84 (19.49)	21 (14.79)	68 (20.12)	88 (16.09)	112 (21.41)	78 (15.18)	47 (12.91)								
	Preparation	72 (16.71)	30 (21.13)	72 (21.3)	59 (10.79)	106 (20.27)	112 (21.79)	68 (18.68)								
	Action	14 (3.25)	5 (3.52)	14 (4.14)	5 (0.91)	23 (4.4)	25 (4.86)	9 (2.47)								
	Maintenance	131 (30.39)	69 (48.59)	105 (31.07)	100 (18.28)	159 (30.4)	223 (43.39)	193 (53.02)								

^a^*F* test (*df*).

^b^Chi-square test (*df*).

^c^N/A: not applicable.

^d^PA: physical activity.

^e^MET: metabolic equivalent.

## Discussion

### Principal Findings

This study investigated how individuals perceive BCTs offered in health messages to support PA. Specifically, we explored which clusters would be identified by individuals’ motivation ratings for messages extracted from the extended message bank of MacPherson et al [18]. LCA identified 7 clusters as the optimal solution. Overall, clusters 7 and 6 showed higher ratings across messages, clusters 5 and 3 showed moderate ratings, and clusters 1 and 4 showed lower ratings. Cluster 2 showed the most variable ratings across messages. However, within each broad rating level, clusters differed in their preferences for specific BCTs. The observed heterogeneity in BCT preferences across clusters may be partly explained by differences in the psychological resources individuals require at different stages of behavior change, as proposed by the BCRM framework.

Clusters 7 and 6 showed generally high ratings across different message types. Cluster 7 was labeled “General receptivity” based on its uniformly high ratings across all BCT types, with no particular preference or aversion (no BCTs exceeding a within-cluster standardized mean of ±0.20). Individuals in this cluster had the highest PA levels and approximately half were in the maintenance stage, suggesting the establishment of stable PA habits. Cluster 6 was labeled “Preference for low-effort boosting” based on its highest rating within the cluster for information about social and environmental consequences (5.3) and its lowest rating within the cluster for behavioral experiments (4.4). This cluster showed generally high ratings across BCT types, but with a clearer preference pattern than cluster 7. Both BCTs fall under boosting in the BCRM framework, but they may differ in the degree of active engagement required. In the BCRM, reflective resources refer to internal cognitive capacities that support behavior change, such as beliefs about consequences, intentions, and decision-making processes. While behavioral experiments (4.4) may require active self-engagement, information about consequences (5.3) may allow individuals to build reflective resources, such as beliefs about consequences and decision-making processes, more passively. This suggests that individuals in cluster 6 preferred less effortful forms of boosting. Approximately half of the individuals were in the maintenance stage, where behavior tends to become habitual and automatic, and the need for further change is often reduced [35]. Therefore, individuals in this cluster may have preferred approaches that build reflective resources, such as beliefs about consequences, through passive receipt of information, rather than those requiring active cognitive engagement, such as deliberate self-monitoring or behavioral experimentation.

Clusters 5 and 3 showed overall moderate ratings across BCT types. Cluster 5 was labeled “Preference for outcome information and goal setting” based on its highest rating for information about social and environmental consequences (5.3) within the cluster, and goal setting (1.3), which exceeded the reference threshold only in this cluster, with no BCTs rated notably low (no BCTs below a within-cluster standardized mean of –0.20). Cluster 3 was labeled “Preference for outcome information and rewards over prompts” based on its highest rating within the cluster for information about social and environmental consequences (5.3), rewards such as reward approximation (14.4), which exceeded the positive reference threshold only in this cluster, and its lowest rating within the cluster for prompts/cues (7.1). In both clusters, more than half of individuals were in the preaction stages (precontemplation to preparation; ≥65%), in which knowledge and insight are considered necessary to motivate behavior change [35]. Furthermore, nudging BCTs (reward approximation and prompts/cues), which activate affective resources without requiring conscious deliberation [34], were responded to differently between the 2 clusters. Therefore, even within the same stage, cluster 5 was characterized by a preference for building reflective resources through outcome information, whereas cluster 3 showed a greater emphasis on activating affective resources in addition to such information.

Clusters 4 and 1 showed overall low ratings across BCT types. Cluster 4 was labeled “Low receptivity with preference for outcome information” based on its highest rating within the cluster for salience of consequences (5.2) and its lowest rating within the cluster for prompts/cues (7.1). This cluster had the highest proportion of individuals in the precontemplation stage and the lowest PA levels among all clusters. At a stage where motivation was low and the benefits of PA were not well recognized, prompts/cues that encourage PA may be less acceptable (eg, “Reminders can be super helpful when we are trying to form new habits. Put a reminder in your phone, a note on your fridge, or get creative!”). Instead, BCTs that can enhance intrinsic motivation toward behavior change, such as providing information about outcomes, may be more effective for this group. Cluster 1 was labeled “Preference for external outcome information without self-engagement” based on its highest rating within the cluster for information about social and environmental consequences (5.3), its lowest rating within the cluster for monitoring of emotional consequences (5.4), and identification of self as role model (13.1), which exceeded the negative reference threshold only in this cluster. A large proportion of individuals in this cluster were in the preaction stage. In this preaction stage, where knowledge and insight are considered necessary to motivate behavior change, individuals in this cluster may have preferred approaches that provide external information about outcomes, rather than those requiring active self-reflection or role-based engagement.

Finally, cluster 2 was labeled “Selective preference for concrete guidance with framing sensitivity” based on its greatest variability in BCT preferences among all clusters. Specifically, this cluster showed the highest rating within the cluster for salience of consequences (5.2) and the lowest rating within the cluster for monitoring of emotional consequences (5.4). With the exception of instruction on how to perform the behavior (4.1), commonly used BCT groups (ie, goals and planning, feedback and monitoring, and social support) did not exceed the positive within-cluster standardized mean reference threshold in this cluster. In addition, both gain- and loss-framed messages exceeded the positive reference threshold only in this cluster, with a slightly stronger preference for gain framing. Approximately half of the individuals were in the maintenance stage, with PA levels similar to cluster 7. From the perspective of prospect theory, gain framing is generally more effective for low-risk preventive behaviors, whereas loss framing is considered more effective for high-risk detection behaviors [54]. Given that many individuals in cluster 2 were already engaging in PA, gain framing may have been slightly more preferred in the context of preventive action. Moreover, cluster 2 had the highest mean age among the clusters, and the messages explicitly referred to conditions more commonly associated with older adults, such as hypertension and diabetes (eg, “Eat more leafy greens and less sugar, and exercise more! These simple actions have been shown to decrease the risk of type 2 diabetes”). Therefore, the self-relevance of the messages may have contributed to the preference for gain-framed messages in cluster 2, although the interaction between age and message framing was not directly tested in this study. Future research should further examine the role of age, including whether the inclusion of age-relevant diseases in messages influences framing effects.

Previous systematic reviews have suggested that behavior change interventions for PA frequently implement the following BCTs: goal setting, social support, self-monitoring of behavior, and instruction on how to perform the behavior [24-31]. Our findings revealed between-cluster differences in the motivation ratings for these common BCTs. For example, goal setting (1.3) exceeded the positive within-cluster standardized mean reference threshold only in cluster 5, whereas it fell below the negative reference threshold only in cluster 2. Instruction on how to perform the behavior (4.1) exceeded the positive reference threshold only in cluster 2. In contrast, self-monitoring of behavior (2.3) fell below the negative reference threshold in clusters 1, 2, and 6, as did social support (3.1) in clusters 1 and 2, and social support (3.2) in cluster 2. These results align with previous findings that frequently used BCTs are not necessarily perceived as helpful or motivational [55,56]. Furthermore, self-monitoring attracts different viewpoints from intervention recipients [57]. Some individuals found diary (manual) recordings of PA repetitive and time-consuming, whereas others found it useful to visualize progress and detect barriers to PA. The effect of self-monitoring on PA is also somewhat mixed, and self-monitoring is suggested to work only when delivered with objective monitoring tools (eg, accelerometers) [58]. Future research must disentangle the discrepancies between the perceived and actual efficacy of self-monitoring considering individual differences because some individuals may perceive it as boring but benefit from it by changing their PA habits.

The findings of this study have several practical implications. When designing a TMI with tailored message delivery, the first decision should be whether the target recipients are open to the TMI. Our results suggest that several clusters rate any message as not motivational. These clusters included a higher proportion of individuals in the preaction stages, suggesting that selecting BCTs that target the resources required at this stage may be effective. The preaction stage is characterized by low motivation for behavior change, with a need for knowledge and insight. Therefore, presenting information about outcomes may be beneficial for building reflective resources for behavior change. In contrast, approaches requiring active self-engagement or providing concrete instructions for action may be less acceptable. Additionally, given the limited reflective resources at this stage, the use of rewards may also be somewhat effective as a supplementary approach. Similarly, practitioners could refer to demographic information when tailoring messages to send. Cluster 2 typically included older people, and this cluster showed relatively higher ratings for both gain- and loss-framed messages, suggesting that age may be a relevant factor in framing preferences, although this was not directly tested in this study. Tailoring variables do not have to be limited to demographic and trait-like characteristics, such as PA and SoC. Fu et al [59] suggested that the key to further facilitating personalization would be for an intervention to assess what BCTs have and have not worked for each individual at an early stage of the intervention. This would clarify the additional support that should be offered to meet individuals’ changing needs over the progress of an intervention, considering that different BCTs would be needed at different stages of intervention.

### Limitations

This study had several limitations. First, we exclusively recruited Japanese-speaking adults with Internet access. Caution should be exercised when interpreting these results in terms of cultural differences and generalizability. Cluster 2 included several older people who showed unique preferences for framed messages. However, these individuals may not be representative of the older population, which is the generation typically facing the digital divide [60]. Furthermore, the potential influence of selection bias should be considered. Because the participants had previously taken part in surveys conducted by our research group [43], the sample might be more motivated, health-aware, and digitally literate than the general population. This may have generally increased positive responses to mHealth interventions and may also have contributed to the relatively high baseline PA levels, as well as the presence of clusters characterized by high responsiveness to messages (eg, cluster 7). Second, motivation was rated cross-sectionally. Therefore, whether each message triggers actual behavior, and not only motivation or intention, remains unclear. While the association between intention and PA is well established, substantial discrepancies have been observed in experimental studies [61]. Therefore, a high level of motivation in response to messages may not necessarily translate directly into increased PA. Additional constructs that strengthen intention (eg, self-regulation and automaticity) have been proposed. Future research should examine, through experimental and longitudinal designs, whether higher motivation leads to engagement in PA or whether it operates through interactions with other factors. Third, standardizing each message for the tone used and resources referred to (eg, national guidelines) was difficult, mainly because we relied on an established message bank. We focused on the BCTs offered in each message as the key dimension of the analysis; however, motivation ratings could be affected by other factors. Our intention was to analyze an existing message bank to leverage individual differences in motivation ratings for tailoring message delivery. However, a future study could use a factorial design to rigorously control for other factors to clarify what (combinations of) factors determine motivation. Furthermore, fatigue and reduced concentration associated with evaluating 83 messages may have influenced participants’ ratings. Fourth, we specified message delivery in the mHealth context, and the participants were instructed to assume that each message was sent by an AI chatbot implemented in a health care app. This was because this study was intended to be the basis for future mHealth TMIs. However, different senders (eg, human health care professionals) may cause the messages to be perceived differently. Beyond the specific delivery context, the text message format itself has inherent limitations. TMIs remain a valuable component of mHealth due to their cost-effectiveness, ease of personalization, and potential to be scaled up as ecological momentary or just-in-time adaptive interventions. However, not all BCTs may be equally suited to this format. For example, self-monitoring typically assumes a bidirectional process in which individuals record behavior and receive feedback accordingly. The messages used in this study merely prompted recording without reproducing this interactive process, which may have contributed to the relatively low motivation ratings for self-monitoring. Future research should therefore examine how BCTs can be effectively combined with interactive and multimodal features of mHealth tools, such as apps with feedback loops and wearable integration, to clarify which BCTs are most suitable for text message-based delivery and which require more dynamic formats. Fifth, the possibility of cluster solutions other than the 7-cluster solution remains. When conducting a latent profile analysis using continuous variables, models with 9 or more clusters failed to converge, preventing a full comparison across solutions. Although the BIC was lowest among the converged solutions for the 8-cluster solution, we were unable to demonstrate the adequacy of the 8-cluster solution. Therefore, the 7-cluster solution was selected based on the BIC and entropy obtained from the LCA. Nevertheless, the identified clusters should be interpreted as a pragmatic summary of response patterns rather than definitive latent subgroups, as more complex solutions may also exist with continuous variables. Further investigation is needed to determine whether the 7-cluster solution can be replicated in other datasets. Sixth, the number of messages per BCT included in the data bank was unbalanced. As the LCA was conducted using dichotomized motivation ratings for each of the 83 individual messages as input variables, BCTs represented by a greater number of messages may have had a greater influence on the cluster patterns. No weighting or adjustment was applied to account for this imbalance, as it reflects the composition of the original message bank. Its potential impact on the clustering results should therefore be considered when interpreting the findings.

### Conclusion

Despite the limitations, we believe that our findings will pave the way for data-driven approaches to tailored health message delivery for enhancing PA. Our analysis identified clusters of individuals who were generally open to any BCTs, and these individuals had typically been active and maintained PA habits. The clusters that did not find the messages to be motivational were generally less active and in the preaction stages. However, they appeared to appreciate information about the consequences of health behaviors, with some also showing a preference for rewards, which may serve as a good starting point should TMIs be offered for inactive individuals. The BCTs that have commonly been used in interventions were not necessarily perceived as motivational. Although we found some individual (or between-cluster) differences, self-monitoring was not consistently rated as motivational across all clusters. This does not mean that self-monitoring and other common BCTs are not effective in promoting PA, but that some care would be appropriate to reduce message recipients’ resistance when introducing these BCTs. To further increase message acceptability, it is important to consider the mechanisms of action [34,62] to address individual needs, which can be communicated more explicitly in each message. In this study, BCRM was used as one perspective on mechanisms of action to interpret the psychological resources underlying preferences for different messages. The mechanisms of action serve as another tailoring dimension along with the BCT taxonomy, while identifying what proximate outcomes (eg, knowledge and skills) should be targeted for individual message recipients. Furthermore, drawing on classification systems grounded in self-determination theory, such as that proposed by Ahmadi et al [63], may help clarify the links between these outcomes and underlying motivational processes. Future research should expand the focus to how BCTs could be contextualized in interventions (eg, specifying proximate outcomes and resolving how they could be achieved) to make TMIs more personalized, cover the taxonomy, and achieve dynamic and process-oriented tailoring.

## Data Availability

The datasets generated or analyzed during this study are available from the corresponding author on reasonable request.

## Appendix

Multimedia Appendix 1 Supplementary tables.

## Abbreviations

BCRM: Behavior Change Resource Model
BCT: behavior change technique
BCW: behavior change wheel
BIC: Bayesian Information Criterion
COM-B: capability, opportunity, motivation-behavior
IPAQ-SF: International Physical Activity Questionnaire-Short Form
LCA: latent class analysis
MET: metabolic equivalent
mHealth: mobile health
PA: physical activity
SoC: stages of change
STROBE: Strengthening the Reporting of Observational Studies in Epidemiology
TMI: text message intervention
