# Supplementary material for: Exploring Response Patterns to Motivational Messages Supporting Physical Activity: Latent Class Analysis
Source: JMIR Hum Factors. 2026 Aug 19;13:e91917. doi: 10.2196/91917 (PMC13539174; doi:10.2196/91917)
Supplement: Supplementary file 1 [file humanfactors_v13i1e91917_app1.docx]

Exploring response patterns to motivational messages supporting physical activity: A latent class analysis

Supplementary materials

Table S1. Additional messages with BCT labels and framing

| Japanese | Message | BCT tags/framing |
| --- | --- | --- |
| 歩行またはそれと同等以上の強度の身体活動を１日60分以上（１日約8,000歩以上に相当）行うことで，体力が強化され健康が増進されます。 | If you engage in walking or equivalent intensity exercise (equivalent to approximately 8000 steps per day) for 60 minutes or more daily, you can improve your fitness and overall health. | Gain;Information about health consequences (5.1) |
| 歩行またはそれと同等以上の強度の身体活動を１日60分以上（１日約8,000歩以上に相当）行わないと，体力が低下し健康を害することになります。 | If you do not engage in walking or equivalent intensity exercise (equivalent to approximately 8000 steps per day) for 60 minutes or more daily, your fitness and overall health will decline. | Loss;Information about health consequences (5.1) |
| 日本では，成人および高齢者に筋トレを週２～３日実施することを推奨しています。筋トレを行うことで筋肉が増え，楽に身体を動かすことができます。 | In Japan, it is recommended that adults and seniors engage in strength training two to three times a week. Strength training increases muscle mass, making it easier to move your body. | Gain;Information about health consequences (5.1);Credible source (9.1) |
| 日本では，成人および高齢者に筋トレを週２～３日実施することを推奨しています。筋トレを行わないと筋肉が増えず，楽に身体を動かすことができなくなります。 | In Japan, it is recommended that adults and seniors engage in strength training two to three times a week. Without strength training, muscles will not grow, making it harder to move your body with ease. | Loss;Information about health consequences (5.1);Credible source (9.1) |
| 厚生労働省のガイドラインでは，息が弾み汗をかく程度の運動を週60分以上行うことを推奨しています。このような運動を実施すると糖尿病や心疾患などの病気を予防することができます。 | The guidelines of The Ministry of Health, Labour and Welfare recommend engaging in at least 60 minutes of aerobic exercise per week. Engaging in this level of exercise can help prevent diseases such as diabetes and heart disease. | Gain;Information about health consequences (5.1);Credible source (9.1) |
| 厚生労働省のガイドラインでは，息が弾み汗をかく程度の運動を週60分以上行うことを推奨しています。このような運動を実施しないでいると糖尿病や心疾患などの病気になってしまいます。 | The guidelines of the Ministry of Health, Labour and Welfare recommend engaging in at least 60 minutes of aerobic exercise per week. Failure to meet this level of activity increases the risk of developing diseases such as diabetes and heart disease. | Loss;Information about health consequences (5.1);Credible source (9.1) |
| 令和２年のスポーツ庁の調査では、週に一回以上、スポーツを実施している人の割合が60％を超えました。このことから、多くの人がスポーツを継続的に楽しんでいるといえます。 | According to a 2020 survey by the Japan Sports Agency, over 60% of people engage in sports at least once a week. This indicates that many people enjoy sports on a regular basis. | Information about emotional consequences (5.6);Credible source (9.1) |
| 令和4年のスポーツ庁の調査では、週に一回以上スポーツを実施している人の割合が年々減少していることがわかりました。このことから、コロナパンデミック以降、スポーツを継続するのが難しいと感じている人が少なからずいることがわかります。 | A 2022 survey by the Japan Sports Agency revealed that the ratio of people engaging in sports at least once a week has been declining year by year. This indicates that since the COVID-19 pandemic, a significant number of people have found it difficult to continue participating in sports. | Information about health consequences (5.1);Credible source (9.1) |
| 運動療法により血糖コントロール・インスリン抵抗性・脂質代謝の改善が得られ、糖尿病を改善できます。運動療法の目標として、運動の頻度は週に150分かそれ以上、週に3回以上、運動強度は中等度（ややきつい）の全身を使った有酸素運動（ウォーキング・ジョギング・水泳など）、運動持続時間は20分以上行うことが勧められています。 | Exercise therapy can improve glycemic control, insulin resistance, and lipid metabolism, thereby improving diabetes. As goals for exercise therapy, it is recommended that the frequency of exercise be 150 minutes or more per week, at least 3 times per week, moderate (somewhat strenuous) full-body aerobic exercise (walking, jogging, swimming, etc.), and exercise duration at least 20 minutes. | Instruction on how to perform the behaviour (4.1);Information about health consequences (5.1);Credible source (9.1) |
| 運動は高血圧症の改善に効果的です。「ややきつい」と感じる程度の強度で，毎日30分以上の運動，または1回につき少なくとも10分以上持続し、合計して1日40分以上運動を試してみましょう。 | Exercise is an effective way to improve hypertension. Try exercising at an intensity that feels “somewhat strenuous” for at least 30 minutes each day, or at least 10 minutes per session, for a total of at least 40 minutes per day. | Instruction on how to perform the behaviour (4.1);Information about health consequences (5.1);Credible source (9.1) |
| 高齢者の方は，歩行またはそれと同等以上の強度の身体活動を１日40分以上行うことを推奨されています（１日約6,000歩以上に相当）。簡単な運動から始めてみましょう。 | It is recommended that older adults walk or engage in physical activity of equivalent or greater intensity for at least 40 minutes per day (equivalent to about 6,000 steps or more per day). Start with simple exercise. | Instruction on how to perform the behaviour (4.1);Credible source (9.1) |

Table S2. Post-hoc tests on the between-cluster differences in age and physical activity (PA)

| Variable | Contrast | Estimate | SE | t value | p value | d | CI |
| --- | --- | --- | --- | --- | --- | --- | --- |
| Age | C2 vs. |  |  |  |  |  |  |
|  | C1 | 4.494 | 1.652 | 2.72 | 0.009 | 0.263 | [0.073, 0.453] |
|  | C7 | 6.909 | 1.689 | 4.09 | < .001 | 0.405 | [0.210, 0.599] |
|  | C3 | 7.057 | 1.707 | 4.134 | < .001 | 0.413 | [0.217, 0.610] |
|  | C4 | 10.112 | 1.608 | 6.289 | < .001 | 0.592 | [0.407, 0.778] |
|  | C6 | 13.307 | 1.618 | 8.222 | < .001 | 0.779 | [0.592, 0.966] |
|  | C5 | 13.882 | 1.615 | 8.593 | < .001 | 0.813 | [0.626, 1.000] |
|  | C1 vs. |  |  |  |  |  |  |
|  | C7 | 2.415 | 1.215 | 1.987 | 0.052 | 0.141 | [0.002, 0.281] |
|  | C3 | 2.564 | 1.24 | 2.067 | 0.045 | 0.150 | [0.008, 0.293] |
|  | C4 | 5.619 | 1.1 | 5.11 | < .001 | 0.329 | [0.203, 0.456] |
|  | C6 | 8.813 | 1.115 | 7.904 | < .001 | 0.516 | [0.387, 0.645] |
|  | C5 | 9.389 | 1.111 | 8.454 | < .001 | 0.550 | [0.422, 0.678] |
|  | C7 vs. |  |  |  |  |  |  |
|  | C3 | 0.149 | 1.29 | 0.115 | 0.908 | 0.009 | [-0.139, 0.157] |
|  | C4 | 3.204 | 1.155 | 2.774 | 0.008 | 0.188 | [0.055, 0.320] |
|  | C6 | 6.398 | 1.169 | 5.471 | < .001 | 0.375 | [0.240, 0.509] |
|  | C5 | 6.974 | 1.165 | 5.984 | < .001 | 0.408 | [0.274, 0.543] |
|  | C3 vs. |  |  |  |  |  |  |
|  | C4 | 3.055 | 1.181 | 2.586 | 0.012 | 0.179 | [0.043, 0.315] |
|  | C6 | 6.250 | 1.196 | 5.228 | < .001 | 0.366 | [0.228, 0.504] |
|  | C5 | 6.825 | 1.191 | 5.728 | < .001 | 0.400 | [0.263, 0.537] |
|  | C4 vs. |  |  |  |  |  |  |
|  | C6 | 3.195 | 1.049 | 3.046 | 0.004 | 0.187 | [0.067, 0.308] |
|  | C5 | 3.770 | 1.044 | 3.611 | 0.001 | 0.221 | [0.101, 0.341] |
|  | C6 vs. |  |  |  |  |  |  |
|  | C5 | 0.575 | 1.06 | 0.543 | 0.617 | 0.034 | [-0.088, 0.155] |
| PA | C7 vs. |  |  |  |  |  |  |
|  | C2 | 8.448 | 5.502 | 1.536 | 0.154 | 0.152 | [-0.042, 0.346] |
|  | C6 | 11.112 | 3.809 | 2.917 | 0.008 | 0.200 | [0.065, 0.334] |
|  | C5 | 16.987 | 3.796 | 4.476 | <.001 | 0.306 | [0.171, 0.440] |
|  | C3 | 17.481 | 4.200 | 4.162 | <.001 | 0.314 | [0.166, 0.463] |
|  | C1 | 19.708 | 3.958 | 4.979 | <.001 | 0.354 | [0.215, 0.494] |
|  | C4 | 28.867 | 3.761 | 7.675 | <.001 | 0.519 | [0.386, 0.652] |
|  | C2 vs. |  |  |  |  |  |  |
|  | C6 | 2.665 | 5.272 | 0.505 | 0.644 | 0.048 | [-0.138, 0.234] |
|  | C5 | 8.540 | 5.262 | 1.623 | 0.137 | 0.154 | [-0.032, 0.339] |
|  | C3 | 9.033 | 5.561 | 1.624 | 0.137 | 0.162 | [-0.034, 0.359] |
|  | C1 | 11.26 | 5.380 | 2.093 | 0.064 | 0.203 | [0.013, 0.392] |
|  | C4 | 20.419 | 5.237 | 3.899 | <.001 | 0.367 | [0.182, 0.552] |
|  | C6 vs. |  |  |  |  |  |  |
|  | C5 | 5.875 | 3.454 | 1.701 | 0.137 | 0.106 | [-0.016, 0.227] |
|  | C3 | 6.368 | 3.894 | 1.635 | 0.137 | 0.115 | [-0.023, 0.252] |
|  | C1 | 8.595 | 3.632 | 2.367 | 0.034 | 0.155 | [0.026, 0.283] |
|  | C4 | 17.754 | 3.416 | 5.198 | <.001 | 0.319 | [0.199, 0.440] |
|  | C5 vs. |  |  |  |  |  |  |
|  | C3 | 0.493 | 3.881 | 0.127 | 0.899 | 0.009 | [-0.128, 0.146] |
|  | C1 | 2.720 | 3.617 | 0.752 | 0.527 | 0.049 | [-0.079, 0.176] |
|  | C4 | 11.879 | 3.401 | 3.493 | 0.001 | 0.214 | [0.094, 0.334] |
|  | C3 vs. |  |  |  |  |  |  |
|  | C1 | 2.227 | 4.04 | 0.551 | 0.643 | 0.040 | [-0.102, 0.183] |
|  | C4 | 11.386 | 3.847 | 2.96 | 0.008 | 0.205 | [0.069, 0.341] |
|  | C1 vs. |  |  |  |  |  |  |
|  | C4 | 9.159 | 3.581 | 2.557 | 0.022 | 0.165 | [0.038, 0.291] |

*Note.* P-values were adjusted for multiple testing using the Benjamini-Hochberg procedure. C = cluster.

Table S3. Post-hoc tests on between-cluster differences in the maintenance stage

| Contrast | Estimate | SE | z value | p value | OR | CI |
| --- | --- | --- | --- | --- | --- | --- |
| C7 vs. |  |  |  |  |  |  |
| C2 | 0.177 | 0.198 | 0.896 | 0.432 | 1.194 | [0.654, 2.179] |
| C6 | 0.387 | 0.138 | 2.813 | 0.006 | 1.473 | [0.969, 2.237] |
| C3 | 0.918 | 0.158 | 5.825 | < .001 | 2.505 | [1.551, 4.043] |
| C5 | 0.949 | 0.142 | 6.701 | < .001 | 2.584 | [1.680, 3.975] |
| C1 | 0.950 | 0.148 | 6.403 | < .001 | 2.585 | [1.647, 4.055] |
| C4 | 1.618 | 0.153 | 10.61 | < .001 | 5.045 | [3.174, 8.020] |
| C2 vs. |  |  |  |  |  |  |
| C6 | 0.210 | 0.190 | 1.104 | 0.333 | 1.233 | [0.692, 2.197] |
| C3 | 0.741 | 0.205 | 3.614 | < .001 | 2.097 | [1.125, 3.908] |
| C5 | 0.772 | 0.193 | 4.001 | < .001 | 2.164 | [1.204, 3.888] |
| C1 | 0.772 | 0.198 | 3.902 | < .001 | 2.165 | [1.186, 3.947] |
| C4 | 1.441 | 0.201 | 7.167 | < .001 | 4.225 | [2.293, 7.783] |
| C6 vs. |  |  |  |  |  |  |
| C3 | 0.531 | 0.147 | 3.601 | < .001 | 1.701 | [1.087, 2.662] |
| C5 | 0.562 | 0.13 | 4.317 | < .001 | 1.754 | [1.181, 2.606] |
| C1 | 0.562 | 0.137 | 4.092 | < .001 | 1.755 | [1.156, 2.664] |
| C4 | 1.231 | 0.142 | 8.672 | < .001 | 3.425 | [2.226, 5.275] |
| C3 vs. |  |  |  |  |  |  |
| C5 | 0.031 | 0.151 | 0.206 | 0.883 | 1.032 | [0.652, 1.632] |
| C1 | 0.032 | 0.157 | 0.200 | 0.883 | 1.032 | [0.640, 1.665] |
| C4 | 0.700 | 0.161 | 4.339 | < .001 | 2.014 | [1.234, 3.290] |
| C5 vs. |  |  |  |  |  |  |
| C1 | 0.000 | 0.141 | 0.002 | 0.998 | 1.000 | [0.651, 1.537] |
| C4 | 0.669 | 0.146 | 4.588 | < .001 | 1.953 | [1.254, 3.040] |
| C1 vs. |  |  |  |  |  |  |
| C4 | 0.669 | 0.152 | 4.391 | < .001 | 1.952 | [1.229, 3.102] |

*Note.* Generalized linear regressions were conducted with clusters as predictors and with the maintenance stage (vs. precontemplation-action stages) as the outcome. P-values were adjusted for multiple testing using the Benjamini-Hochberg procedure. C = cluster.
